# The Long Terminal Repeats of ERV6 Are Activated in Pre-Implantation Embryos of Cynomolgus Monkey

**DOI:** 10.3390/cells10102710

**Published:** 2021-10-09

**Authors:** Kui Duan, Chen-Yang Si, Shu-Mei Zhao, Zong-Yong Ai, Bao-Hua Niu, Yu Yin, Li-Feng Xiang, Hao Ding, Yun Zheng

**Affiliations:** 1 Faculty of Environmental Science and Engineering, Kunming University of Science and Technology, Kunming 650500, China; duank@lpbr.cn (K.D.); sicy@lpbr.cn (C.-Y.S.); zhaosm@lpbr.cn (S.-M.Z.); aizy@lpbr.cn (Z.-Y.A.); niubh@lpbr.cn (B.-H.N.); yiny@lpbr.cn (Y.Y.); xlflotus@aliyun.com (L.-F.X.); haoding2020@163.com (H.D.); 2 Faculty of Life Science and Technology, Kunming University of Science and Technology, Kunming 650500, China; 3 Yunnan Key Laboratory of Primate Biomedical Research, Kunming University of Science and Technology, Kunming 650500, China; 4 State Key Laboratory of Primate Biomedical Research, Institute of Primate Translational Medicine, Kunming University of Science and Technology, Kunming 650500, China; 5 Yunnan Provincial Academy of Science and Technology, Kunming 650500, China

**Keywords:** MacERV6-LTR1a, primitive endoderm, trophectoderm, epiblast, cynomolgus monkey, embryos, *ESRRB*

## Abstract

Precise gene regulation is critical during embryo development. Long terminal repeat elements (LTRs) of endogenous retroviruses (ERVs) are dynamically expressed in blastocysts of mammalian embryos. However, the expression pattern of LTRs in monkey blastocyst is still unknown. By single-cell RNA-sequencing (seq) data of cynomolgus monkeys, we found that LTRs of several ERV families, including MacERV6, MacERV3, MacERV2, MacERVK1, and MacERVK2, were highly expressed in pre-implantation embryo cells including epiblast (EPI), trophectoderm (TrB), and primitive endoderm (PrE), but were depleted in post-implantation. We knocked down MacERV6-LTR1a in cynomolgus monkeys with a short hairpin RNA (shRNA) strategy to examine the potential function of MacERV6-LTR1a in the early development of monkey embryos. The silence of MacERV6-LTR1a mainly postpones the differentiation of TrB, EPI, and PrE cells in embryos at day 7 compared to control. Moreover, we confirmed MacERV6-LTR1a could recruit Estrogen Related Receptor Beta (ESRRB), which plays an important role in the maintenance of self-renewal and pluripotency of embryonic and trophoblast stem cells through different signaling pathways including FGF and Wnt signaling pathways. In summary, these results suggest that MacERV6-LTR1a is involved in gene regulation of the pre-implantation embryo of the cynomolgus monkeys.

## 1. Introduction

**Figure 1:**
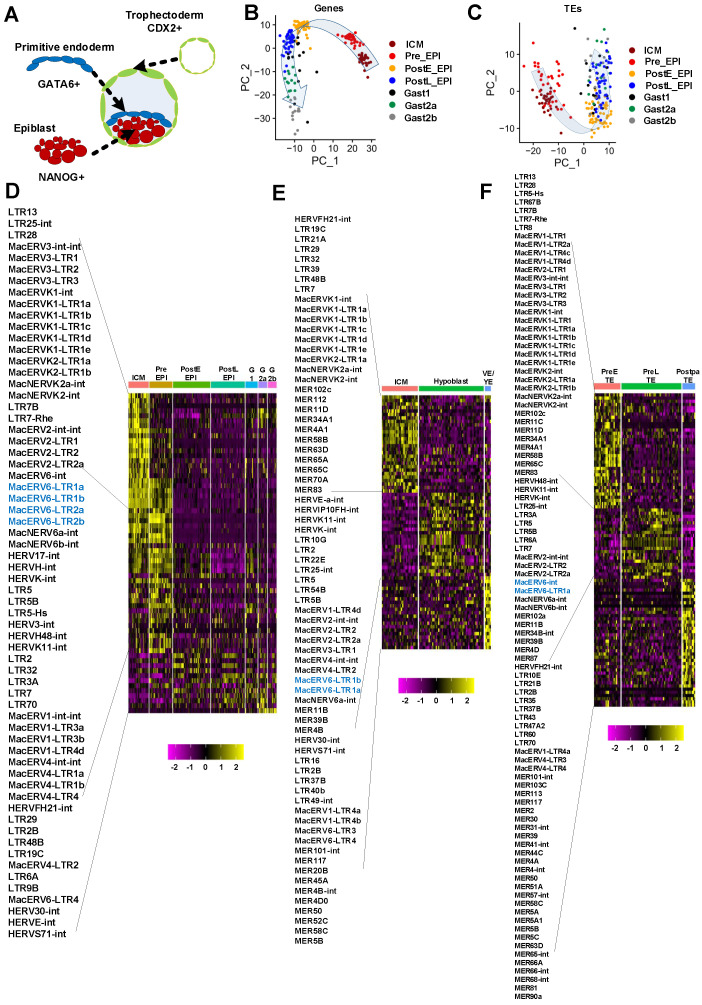
The dynamic expression of LTR elements during the development of *Macaca fascicularis* embryo from blastocysts to the E17 gastrulation stage. (**A**) Schematic view of three lineages of *Macaca fascicularis* blastocyst. (**B**) Principal component analysis with gene expression levels during *Macaca fascicularis* epiblast development with Seurat. (**C**) Principal component analysis with expression levels of transposable element (TEs) during *Macaca fascicularis* epiblast development. (**D**) Heatmap of LTR elements expression during epiblast development. The cell type include inner cell mass (ICM) (*n* = 30), pre-implantation epiblasts (PreEPI) (*n* = 34), post-implantation early EPI (PostEEPI) (*n* = 55), post-implantation late EPI (postLEPI) (*n* = 50), gastrulating cells (G1) (*n* = 18), (G2a) (*n* = 13), and (G2b) (*n* = 13) stages. (**E**) Heatmap of LTR elements exoression during primitive endoderm development. The cell type include inner cell mass (ICM) (*n* = 30), Hypoblast (also named PrE) (*n* = 54), visceral/yolk-sac endoderm (VE/YE) (*n* = 5). (**F**) Heatmap of LTR elements expression during trophectoderm (TrB) development. The cell type include pre-implantation early trophectoderm (PreETE) (*n* = 23), pre-implantation late TE (PreLTE) (*n* = 52), post-implantation parietal trophectoderm (PostpaTE) (*n* = 11).

Mammalian embryogenesis begins with a zygote that develops into a morula, followed by the formation of a blastocyst. At this stage, blastomeres undergo first lineage segregation, giving rise to the trophectoderm (TrB) and inner cell mass (ICM) [1,2,3,4]. As the embryo implants, second lineage segregation occurs and epiblast cells (EPI) in the blastocyst develop into the embryo proper and the amnion [5,6,7], whereas cells of the TE and primitive endoderm (PrE) of the ICM generate the placenta [8] and yolk sac [4,9,10], respectively (Figure 1A). During pre-implantation, the pluripotent development of EPI is transited from a naive to a primed state, and hypoblast cells lose pluripotent genes to generate visceral/yolk-sac endoderm [11,12,13]. The precise transcriptional regulation of EPI, PrE, and TrB is crucial for embryo development of pre-implantation in human and mouse [1,14,15]. Furthermore, different mechanisms were noticed in different species. For example, fibroblast growth factor/extracellular signal-regulated kinase (FGF/ERK) signaling is essential for mouse hypoblast specification. However, previous research has shown that FGF/ERK signaling is not the key factor for human hypoblast specification [16,17].

Endogenous retroviruses (ERVs) are transposable elements that have high copy numbers in mammalian genomes [18,19,20,21,22,23,24,25]. Complete ERVs are flanked by two long terminal repeats (LTRs) that recruit transcription factors (TFs) to regulate the expression of ERVs and nearby genes [26,27,28]. In recent years, the role of LTRs in embryonic cell fate specification and determination has received increasing attention [29,30,31,32]. Since more than 80% of LTRs are located in open chromatin regions in early-stage embryos [33], a large number of LTR elements are dynamically transcribed in early human and mouse embryos [22,29,34,35,36]. For example, many transcripts in two-cell human embryos are initiated by LTR elements [29,37,38], which regulate long non-coding RNAs (lincRNAs) such as HERVH-LTR7 to maintain the naive state of human embryonic stem cells [22,39]. Furthermore, HERVH-LTR7 regions recruit pluripotency factors, including *OCT4*, *SOX2*, and *NANOG* to participate in cell type-specific gene regulatory networks [24,40,41]. Specifically, human embryonic stem cells (hESCs), similar to EPIs at post-implantation stages, may be converted to pre-implantation stage EPI-like cells when blastocyst-specific LTR7 elements are activated [34]. In contrast, the epigenetic mediator, the KRAB-ZFP/KAP1 system, has the ability to silence LTR elements activation in post-implantation embryos in human and mouse [33,34,37,42]. These studies exemplify that specific LTR elements act as crucial mediators in the early embryos of human and mouse. Although many LTR elements have been identified and dynamically expressed in early embryos [24,34], certain stage-specific LTR families have not yet been systematically investigated, especially in the implantation stage of embryos. Moreover, the molecular mechanisms underlying the temporal development of monkey pre-implantation embryos remain unclear [11].

Monkeys are important model species for studying human embryo development because of their high similarity to the human reproduction system [43,44,45]. Here, we examined the transcriptional dynamics of LTR elements in monkeys. Then, we knocked down MacERV6-LTR1a and observed differentiation was postponed in embryonic cells compared to control in the pre-implantation stage. We also validated that MacERV6-LTR1a could recruit ESRRB, which plays an important role in the maintenance of self-renewal and pluripotency of embryonic and trophoblast stem cells through different signaling pathways including FGF signaling pathway and Wnt signaling pathways [46,47,48,49,50,51]. These results suggest that MacERV6-LTR1a is involved in the regulations of the temporal development of early TrB, EPI, and PrE cells in monkey embryos.

## 2. Materials and Methods

### 2.1. Animal Ethics

Female cynomolgus monkeys (*Macaca fascicularis*), ranging from 5 to 8 years and weighing 4–6 kg were used in this study. All animals were housed at Yunnan Key Laboratory of Primate Biomedical Research and individually bred in an American standard cage in a light/dark cycle of 12 h/12 h. All animal procedures were approved in advance by the Institutional Animal Care and Use Committee, and protocols were performed in accordance with the Assessment and Accreditation of Laboratory Animal Care International for the ethical treatment of primates.

### 2.2. Calculation of the Expression Levels of LTR Elements and Genes

The 390 single cell RNA-sequencing (scRNA-seq) data relating to monkey pre- and post-implantation embryos were downloaded from previously published studies (GEO (Gene Expression Omnibus): GSE74767) [52]. RNA-seq reads were filtered by fast quality control (QC) procedures and adaptors cut using Cutadapt software (v1.11). After this, sequences were mapped to the *Macaca fascicularis* genome (macFas5) using HISAT2 software. Then, we quantified transposable element (TEs) and gene expression of single-cell sequencing data using the scTE software [53].

The single cells of monkey embryos were prepared according to smart-seq2 protocol, and sequenced with 2 × 150 base pair (bp) end method using the HiSeq X Ten platform (Illumina, San Diego CA, USA). Library construction and sequencing were performed by Annoroad Gene Technology (http://www.annoroad.com/, accessed on 28 September 2021). The sequencing reads were mapped against the *Macaca fascicularis* genome (macFas5) using HISAT2 (v2.2.1) software. Then, we quantified transposable element (TEs) and gene expression of single-cell sequencing data using the scTE software as before.

### 2.3. Oocyte Collection and In Vitro Fertilization

Superovulation, oocyte collection, and fertilization procedures were previously described [54]. In short, 10 healthy female cynomolgus monkeys aged 5–8 years with regular menstrual cycles were selected as oocyte donors for superovulation. This was performed by intramuscular injection with recombinant human follitropin-α (rhFSH) (GONAL-F, Merck Serono, Darmstadt, Germany) for 8 days. Then, recombinant human chorionic gonadotropin-α (rhCG) (OVIDREL, Merck Serono, Darmstadt, Germany) was injected on day 9. Oocytes were collected by laparoscopic follicular aspiration approximately 32–35 h after rhCG administration. Follicular contents were added to HEPES-buffered Tyrode’s albumin lactate pyruvate (TALP) medium containing 0.3% bovine serum albumin (BSA) (Sigma-Aldrich, Saint-Louis, MO, USA) at 37 °C. Oocytes were stripped of cumulus cells by pipetting after a brief exposure (<1 min) to hyaluronidase (0.5 mg/mL) in TALP-HEPES to visually select nuclear-based mature metaphase II (MII; first polar body present) oocytes. The mature oocytes were immediately subjected to intracytoplasmic sperm injection and then cultured in Connaught Medical Research Laboratories (CMRL) -1066 media (Thermo Fisher Scientific, Waltham, MA, USA) containing 10% fetal bovine serum (FBS, Gibco, USA) at 37 °C in 5% CO${}_{2}$. Fertilization was confirmed by the presence of a second polar body and two pronuclei. Zygotes were then cultured in chemically defined hamster embryo culture medium-9 containing 10% FBS at 37 °C in 5% CO${}_{2}$ to allow embryo development. The culture medium was replaced every other day until the blastocyst stage.

### 2.4. Lentivirus Production and Purification

293T cells in DMEM + 10% FBS + 1% NEAA were split into 6 × 10 cm dishes (3 × 10${}^{6}$ cells/dish) and incubated overnight at 37 °C in 5% CO${}_{2}$. Evolutionary analyses were conducted in MEGAX [55]. The psicoR-EF1A-GFP-shRNA-scramble, psicoR-EF1A-GFP-shRNA-109, psicoR-EF1A-GFP-shRNA-149, and psicoR-EF1A-GFP-shRNA-346 plasmids were prepared according to Jacks laboratory protocols (http://web.mit.edu/jacks-lab/protocols/pSico.html, accessed on 28 September 2021) (Table S1). The psicoR-EF1A-GFP contain a GFP gene driven by EF1A promoter. Therefore, GFP expression could be used as a signal of successful transfection of the plasmid. Per 1 × 10 cm dish, 11 μg shRNA plasmids, and two lentiviral packaging plasmids (3 μg of pMD2.g, and 8 μg of psPAX2) were used. Plasmids were mixed and added to 0.25 M CaCl${}_{2}$ (500 μL), mixed gently in 500 μL BBS solution, incubated at room temperature for 15 min, and added to 293T cells (3 × 10${}^{6}$ cells). Approximately 48 h later, the supernatants were collected, sieved through a 0.45-μm filter (Millipore, Sigma-Aldrich, Saint-Louis, MO, USA), and centrifuged (Beckman, Brea, CA, USA) at 100,000× *g* for 2 h at 4 °C. Viral pellets were then resuspended in 50 μL PBS (pH 7.2–7.4) and infection titers were determined using the gradient dilution method. Simply, a 10-fold serial dilution of the lentivirus solution was added to a 96-well plate (1 × 10${}^{5}$ 293T cells/well) and GFP fluorescence was observed 48 h after infection.

### 2.5. Isolation of Green Fluorescent Protein (GFP) Positive Single Cell and scRNA-Seq

Embryos were dissected into single cells in 0.25% trypsin (Thermo Fisher Scientific, Waltham, MA, USA), washed in Dulbecco’s Phosphate-Buffered Saline (DPBS) (Thermo Fisher Scientific, Waltham, MA, USA), and aspirated using a Pasteur pipette under a dissecting microscope. GFP-positive embryonic cells were collected for gene expression analysis. The synthesis and amplification of full-length cDNAs were performed using the Smart-seq2 protocol [56]. Reverse transcription reactions and pre-amplification steps were performed using SuperScript II (Thermo Fisher Scientific, Waltham, MA, USA) and KAPA HiFi HotStart ReadyMix (KAPA Biosystems, Sigma-Aldrich, Saint-Louis, MO, USA), respectively. The cDNA quality was evaluated using the Bioanalyzer 2100 instrument. Library construction and sequencing were performed by Annoroad Gene Technology. GFP-positive cells were first screened using our previously described PCR method using primers for *GAPDH*. Then, MacERV6-LTR1a expression in GFP positive cells was determined by qRT-PCR and it was performed with iTaq™ universal SYBR Green supermix (Biorad, Hercules, CA, USA) (Table S2). In total, we used 166 single cells to perform pair-end sequencing on the Illumina HiSeq X-Ten platform.

### 2.6. Single-Cell Cluster Analysis

The output of single-cell gene counts was used to create objects using the Seurat package (v3.0) [57]. Principal component analysis (PCA) dimensional reduction techniques were used to visualize gene expression and perform cluster analyses based on the principal components (PCs) input. The VinPlot tool was used to show the probability distribution of marker gene expression across clusters. Differentially expressed genes (DEGs) between different cell types were detected by FindMarkers function of Seurat, test.use with DESeq2 (1.32.0) [58]. *p*-value < 0.05 and min.pct = 0.1 were used to denote significant differences in gene expression. Gene ontology (GO) and Kyoto Encyclopedia of Genes and Genomes (KEGG) pathway analyses of DEGs were conducted using the Metascape website (https://metascape.org/, accessed on 28 September 2021) [59]. GO terms with a *p*-value < 0.05 were defined as significantly enriched.

### 2.7. TF Binding Site Predictions and Dual Luciferase Expression Assays

We first identified motifs that potentially bound to TFs based on conserved nucleotide sequences of MacERV6-LTR1a using MEME algorithm [60]. We then predicted candidate TFs recruited by MacERV-LTR1a based on potential motifs using Tomtom algorithm [61]. We performed Dual-Glo luciferase assays (Promega, Madison WI, USA) to estimate the regulatory potential of MacERV6-LTR1a. We used 293T cells (ATCC CRL-3216) grown in DMEM (Thermo Fisher Scientific, Waltham, MA, USA) supplemented with 10% FBS (Thermo Fisher Scientific, Waltham, MA, USA) and 1% non-essential amino acids (NEAA) (Thermo Fisher Scientific, Waltham, MA, USA). The MacERV6-LTR1a_39 element sequence that contained multiple pluripotency TF-binding sites has cloned into PGL3-basic vector and driven firefly luciferase activity (named pERV6-LTR1a_39). Then, pERV6-LTR1a_39 and pRL-TK vector (expressing renilla luciferase) (Promega, Madison WI, USA) were co-transfected into 293T cells using Lipofectamine 2000 transfection reagent (Thermo Fisher Scientific, Waltham, MA, USA) (2 μL for 1 μg plasmid DNA). All transfections were performed in 24-well plates (Corning, Corning NY, USA). After 24–48 h, luciferase expression levels were processed according to the Dual-Glo reporter assay protocol (Promega, Madison WI, USA). We performed each luciferase experiment in triplicate and repeated each experiment three times. Luciferase fold change was estimated as the ratio of firefly and renilla luciferase for pERV6-LTR1a_39, and then normalized to the empty vector (pGL3 plasmid with no MacERV6-LTR1a_39 insert). We also individually cloned the coding sequences of the genes, *ESRRB*, *KLF4*, *POU5F1*, *SOX2*, *SMAD3*, and *HNF4A* into the PM2 expression vector (pEASY-Blunt M2 Expression Kit, TransGen Biotech, Beijing, CHINA) (Table S2). Furthermore, we synthesized the nucleotide sequences of MacERV6-LTR1a_39 which ESRRB binding site had mutated. Furthermore, then, cloned that sequences into the PGL3-basic vector (named pmERV6-LTR1a_39). The firefly and renilla luciferase fluorescence intensity was measured using a GENios (TECAN, Männedorf, Switzerland). Statistical parameters, including statistical analyses, statistical significance, and n values were reported in figure legends. For significance analysis, Student’s *t*-test was used to indicate a * *p*-value < 0.05 or ** *p*-value < 0.01.

### 2.8. Immunofluorescence

Monkey embryos at day 7 were fixed in 4% paraformaldehyde and 0.1% polyvinyl pyrrolidone (PVP) in PBS (pH 7.2–7.4) for 20 min at room temperature. They were then washed three times in PBS and incubated with 0.3% Triton X-100 and 0.1% PVP for 30 min at room temperature. After washing three times in PBS, embryos were blocked for 2 h in blocking buffer (3% BSA + 10% FBS + 0.1% PVP) and separately incubated overnight at 4 °C with the following primary antibodies: chicken anti-GFP (Abcam, ab13970, 1:500), mouse anti-Oct4 (Santa Cruz, SC5279, C-10, 1:400), and goat anti-Gata6 (R&D Systems, AF1700, polyclonal, 1:400). Embryos were washed three times in PBS/0.05% Tween-20 and incubated with the following secondary antibodies for 2 h at room temperature Alexa Fluor 488 Donkey Anti-Chicken IgY† (IgG) (H + L) (Jackson Immunoresearch, 703-545-155, 1:500), Alexa Fluor 568 donkey anti-mouse IgG Thermo Fisher Scientific, Waltham, MA, USA A-10037, 1:600), and Alexa Fluor 647 donkey anti-goat IgG (Thermo Fisher Scientific, Waltham, MA, USA, A-21447, 1:600). DAPI (Roche Life Science, Basel, Switzerland, 10236276001, 1:1000) was used to stain nuclei.

## 3. Results

### 3.1. Temporal Expression of LTR Elements during Monkey Embryo Development

Implantation is an important event during embryo development. To understand the process, we analyzed the dynamic expression of transposable elements (TEs) in pre- and post-implantation embryos of cynomolgus monkeys based on publicly available scRNA-seq data for *Macaca fascicularis* (GEO accession: GSE74767) which covered embryonic EPI from blastocyst to E17 gastrulation-stages [52]. These scRNA-seq data including ICM (*n* = 30), pre-implantation EPI (pre-EPI, *n* = 34), early post-implantation EPI (postE-EPI, *n* = 55), late post-implantation EPI (postL-EPI, *n* = 50), and three gastrulation cell types (Gast1, *n* = 18, Gast2a, *n* = 13, and Gast2b, *n* = 13). In agreement with gene expression profiles [52], EPI cells can be separated clearly into pre-implantation EPI (ICM and pre-EPI) and post-implantation EPI (postE-EPI, postL-EPI, and gastrulation cells) by expression levels of transposable elements in PCA analysis (Figure 1B,C), showing that the expression of transposable element experienced a dramatic change during implantation. Then, we were particularly interested in LTR elements and found that many LTR elements were dynamically expressed in different stages of three lineages cell types. Firstly, there are 52 LTRs specifically activated in pre-implantation stages and others 13 LTR expressed in post-implantation (Figure 1D). We also observed that the LTR elements of several families, LTR5 (LTR5, LTR5B and LTR5-Hs), LTR7 (LTR7, LTR7B and LTR7-Rhe), and MacERV6-LTR (MacERV6-LTR1a, MacERV6-LTR1b, MacERV6-LTR2a and MacERV6-LTR2b) were specifically activated at ICM and Pre-EPI stages and found that MacERV6-LTRs were highly expressed at Pre-EPI stages, but all of LTRs above were rapidly depleted after implantation (Figure 1D). Of these LTR elements, the LTR7 (LTR7, LTR7B and LTR7-Rhe) and LTR5 (LTR5, LTR5B and LTR5-Hs) were previously reported as templates for initiating stage-specific transcripts in human embryonic cells [34].

**Figure 2:**
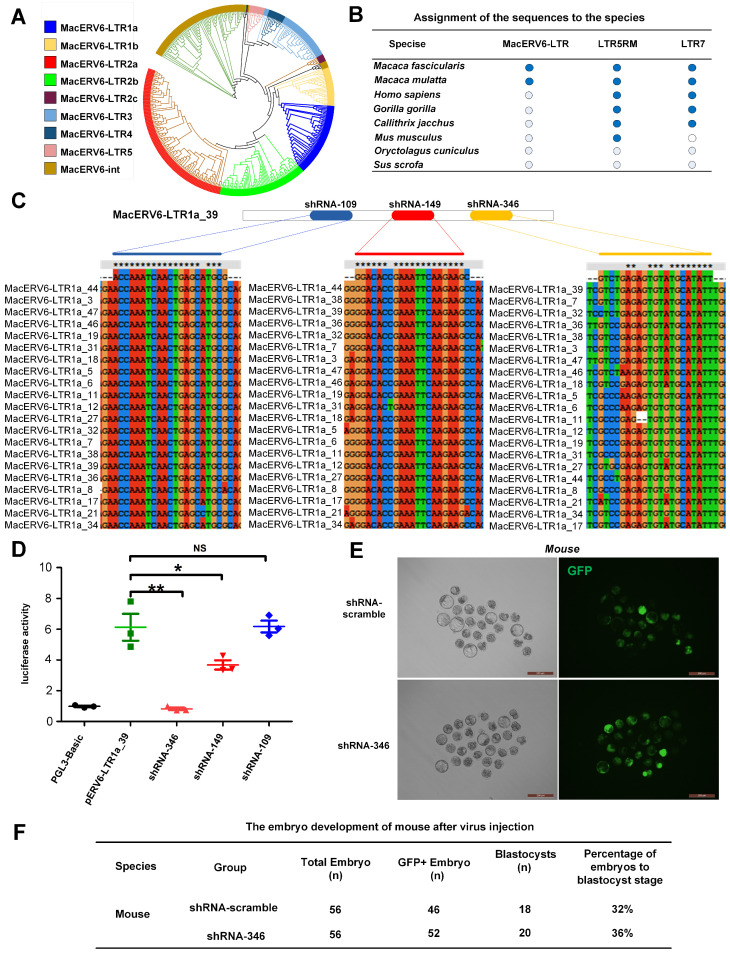
MacERV6-LTR1a knockdown in pre-implantation monkey embryos. (**A**) Phylogenetic tree based on the MacERV6 family sequences. (**B**) The conservation of MacERV6-LTRs, LTR5RM, and LTR7 in selected mammals. The sequences of MacERV6-LTRs, LTR5RM, and LTR7 were aligned to the genomes of different species. Blue circles represent there is the annotated sequence in the genome and white circles are reverse. (**C**) The schematic view of shRNAs, including shRNA-109 (psicoR-EF1A-GFP-shRNA-109), shRNA-149 (psicoR-EF1A-GFP-shRNA-149) and shRNA-346 (psicoR-EF1A-GFP-shRNA-346), targeting MacERV6-LTR1a_39 sites and the conserved sequences of MacERV6-LTR1a. (**D**) The knockdown efficiency of shRNAs targeting MacERV6-LTR1a. PGL3-basic, control plasmid with no promoter. pERV6-LTR1a_39, luciferase reporter was driven by the MacERV6-LTR1a_39 element. pERV6-LTR1a_39 plasmid was co-transfected with shRNA plasmids (shRNA-346, shRNA-149, shRNA-109) into 293T cells. Data were presented as the mean ± standard deviation (SD), (*n* = 3). * *p* < 0.05, ** *p* < 0.01. NS, not significant. Student’s *t*-test. (**E**) GFP signals in mouse embryos that were treated with shRNA-scramble and shRNA-346 lentivirus, separately. Scale bar = 200 μm. (**F**) The development efficiency of mouse embryo after injection with shRNA-scrambled and shRNA-346 lentivirus separately.

To further evaluate whether MacERV6-LTR expression is stage-specific in other lineages, we analyzed its temporal expression in primitive endoderm development (ICM (*n* = 30), Hypoblast (*n* = 54), VE/YE (*n* = 5)). We noticed that MacERV6-LTR (MacERV6-LTR1a, MacERV6-LTR1b) were activated in the Hypoblast cell (Figure 1E). Meanwhile, we evaluated MacERV6-LTR expression in trophectoderm development (PreETE (*n* = 23), PreLTE (*n* = 52), PostpaTE (*n* = 11)) and found that MacERV6-LTR (MacERV6-LTR1a, MacERV6-int) were specifically expressed in the PreLTE stage of pre-implantation embryos (Figure 1F), suggesting that MacERV6-LTR may have specific functions during monkey embryo development. The function of the MacERV6-LTR in pre-implantation embryos is obscure. We selected MacERV6-LTR1a as a family representative for further exprimental validation, because MacERV6s have as many as eight different types of LTRs (Figure 2A).

### 3.2. MacERV6-LTR1a Knockdown in Cynomolgus Monkey Embryos

In order to further understand the conservation of MacERV6-LTRs in various species, we aligned the sequences of MacERV6-LTRs, and other two control (LTR5RM and LTR7) to the genomes of different species, and found that MacERV6-LTRs specifically exist in *Macaca fascicularis* and *Macaca mulatta* (Figure 2B). Previous research suggests that several transcripts in two-cell embryos are initiated by LTRs derived from endogenous retroviruses [29]. Then, we knocked down MacERV6-LTR1a expression to investigate the potential functions of MacERV6-LTR1a during monkey embryo development. Firstly, we designed three shRNAs (shRNA-109, shRNA-149 and shRNA-346) based on conserved regions in the MacERV6-LTR1a, and one scramble shRNA (shRNA-scramble) as the control (Figure 2B,C). Knockdown efficiency was verified using the MacERV6-LTR1a-driven luciferase assay. We observed the luciferase activity has been significantly knocked down by shRNA-149 and shRNA-346 plasmids in 293T cells (Figure 2D). Secondly, the shRNA-346 and shRNA-scramble were selected for virus packaging. To evaluate lentivirus toxicity, 50–100 pL virus (shRNA-346) suspensions expressing shRNA-346 or the shRNA-scrambled control were injected into the perivitelline space of mouse one-cell embryos. GFP fluorescence signals were observed in 90% of embryos at 3–5 days after injection (Figure 2E). No differences in development efficiency were observed in embryos, suggesting that these viral reagents exerted no toxicity on embryo development (Figure 2F).

**Figure 3:**
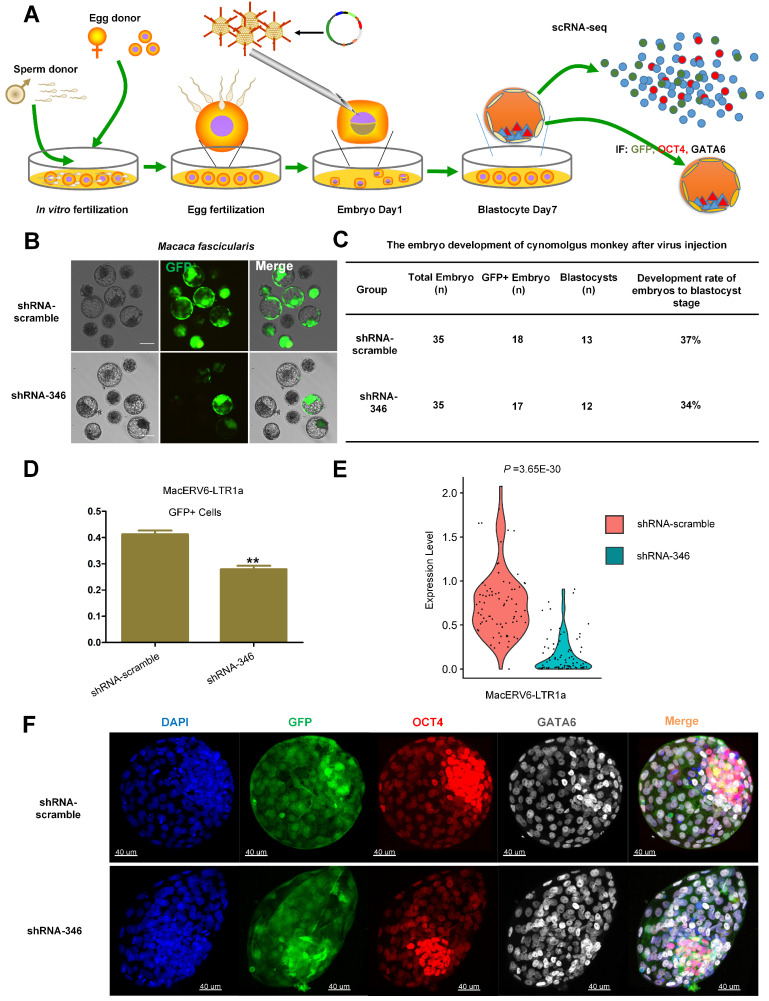
The potential functions of MacERV6-LTR1a in preimplantation cynomolgus monkey embryos. (**A**) Schematic representation of MacERV6-LTR1a functional assays during embryonic development. The mature oocytes were immediately subjected to intracytoplasmic sperm injection and then cultured in Connaught Medical Research Laboratories (CMRL) -1066 media containing 10% fetal bovine serum at 37 °C in 5% CO${}_{2}$. Fertilization was confirmed by the presence of a second polar body and two pronuclei. Zygotes were then cultured in chemically defined hamster embryo culture medium-9 containing 10% FBS at 37 °C in 5% CO${}_{2}$ to allow embryo development. The lentivirus of shRNA-346 and shRNA-scramble were injected at Day 1 of monkey embryos. The culture medium was replaced every other day until the blastocyst stage at Day 7. The embryos were performed Immunofluorescence (IF) in part **F** and scRNA-seq. (**B**) The GFP signals in *Macaca fascicularis* blastocysts with injection of shRNA-scrambled or shRNA-346 lentivirus. Scale bar = 200 μm. (**C**) Blastocyst development efficiency in cynomolgus monkey embryos treated with shRNA-scrambled or shRNA-346 lentivirus. (**D**) The knockdown efficiency of MacERV6-LTR1a in embryonic GFP positive cells treated with shRNA-scramble (*n* = 30 cells) or shRNA-346 (*n* = 30 cells) lentivirus. Quantitative RT-PCR was used to evaluate MacERV6-LTR1a expression. ** *p* < 0.01, Student’s *t*-test. (**E**) Vlnplot showing the expression of MacERV6-LTR1a in cynomolgus monkey embryonic cells at day 7. shRNA-346 (*n* = 86), shRNA-scramble (*n* = 80). *p*-value was evaluated with DEseq2. (**F**) Representative embryo staining images at day 7: GFP (green), OCT4 (red), GATA6 (white), and DAPI (blue). shRNA-scramble (*n* = 3 embryos), shRNA-346 (*n* = 3 embryos). Scale bar = 40 μm.

To further assess the functions of MacERV6-LTR1a in *Macaca fascicularis* embryos, we injected shRNA-346 or the shRNA-scrambled lentivirus into fertilized one-cell cynomolgus monkey embryos (Figure 3A,B). A similar development efficiency of blastocysts were observed in embryos infected by these reagents (Figure 3C). We verified the knockdown efficiency in GFP-positive single cells of embryonic blastocysts on day 7 using qRT-PCR. MacERV6-LTR1a expression was significantly knocked down in shRNA-346-treated cells compared with the shRNA-scramble treated embryonic cells (Figure 3D). Single-cell transcriptome analyses also demonstrated the knockdown efficiency (Figure 3E). Meanwhile, we stained embryos to evaluate the phenotypes on day 7 with specific lineage markers, OCT4 for pre-EPI and GATA6 for hypoblasts to analyze cell fates. However, no significant differences were observed in OCT4 EPI and GATA6 hypoblast development at the whole embryonic level in both embryo groups (Figure 3F).

### 3.3. Transcriptomic Changes after MacERV6-LTR1a Knockdown

**Figure 4:**
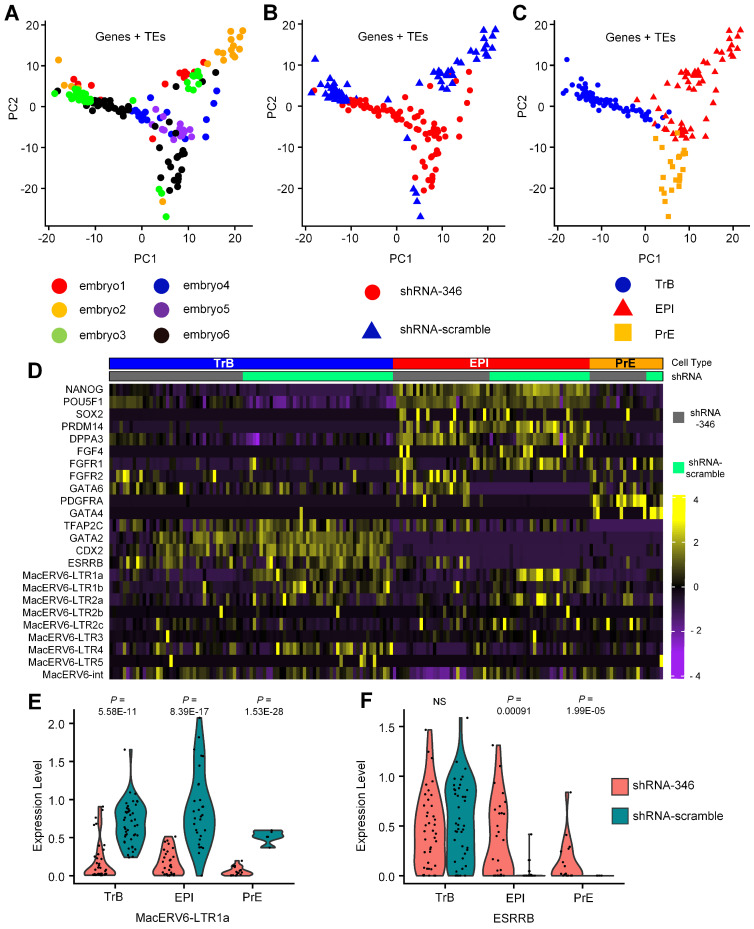
Transcriptional changes in shRNA-346- and shRNA-scramble-treated cynomolgus monkey embryos at day 7. (**A**–**C**) Principal component analysis (PCA) of single cells of embryos according to the expression of genes and TEs. (**A**) The distribution of single cells from six embryos, of which three were the control group (treated with shRNA-scramble, embryo1, embryo2, embryo3) and three were the experimental group (treated with shRNA-346, embryo4, embryo5, embryo6). (**B**) The distribution of single cells of embryos treated with shRNA-scramble (blue triangle) or shRNA-346 (red circle). shRNA-346 represents embryonic cells infected by shRNA-346 lentivirus, whereas shRNA-scramble represents embryonic cells infected by shRNA-scramble control lentivirus. (**C**) Single cells of embryos were annotated as three lineages: TrB, EPI, and PrE, according to the expression of the marker gene in part **D**. (**D**) Heatmap of selected marker genes. *NANOG*, *OCT4*, *PRDM14*, and *DPPA3* for epiblast (EPI), *GATA4* and *PDGFRA* for primitive endoderm/hypoblast (PrE), *CDX2* and *GATA2* for trophectoderm (TrB). (**E**) Vinplots of MacERV6-LTR1a expression in TrB, EPI, and PrE cells that were treated with shRNA-346 and shRNA-scramble lentivirus. (**F**) Vinplots of ESRRB expression in TrB, EPI, and PrE cells that were treated with shRNA-346 and shRNA-scramble lentivirus. NS, not significant. Furthermore, see Figure S2.

To further explore the functions of MacERV6-LTR1a in monkey embryos, GFP positive single cells were collected from blastocysts which were treated with shRNA-346- and the shRNA-scramble at day 7. We performed scRNA-seq using the Smart-seq2 protocol [56]. PCA analyses categorized these cells into three groups based on lineage marker expression (Figure 4A–C). *CDX2*, *GATA2* and *ESRRB* are highly expressed in trophoblast (TrB) populations, along with intermediate *GATA6* expression. Meanwhile, *NANOG*, *OCT4*, *PRDM14*, *DPPA3*, *FGF4*, *FGFR1* and *FGFR2* are heterogeneously expressed in EPI (Pre-implantation epiblast) population. *GATA4* and *PDGFRA* are enriched in primitive endoderm (also named hypoblast or PrE) cluster, along with intermediate *GATA6* expression (Figure 4D). The presence of TrB, PrE, and EPI populations indicated that monkey embryos at day 7 exhibit the segregations of the three lineage. We observed the shutdown state of MacERV6-LTR1a in three lineages cell types, and also found MacERV6-LTR1b, MacERV6-LTR2a and MacERV6-LTR4 were knocked down when treating with shRNA-346 (Figure 4D,E). Interestingly, we observed that the expression of *ESRRB* has significantly changed when treating with shRNA-346 (Figure 4F).

**Figure 5:**
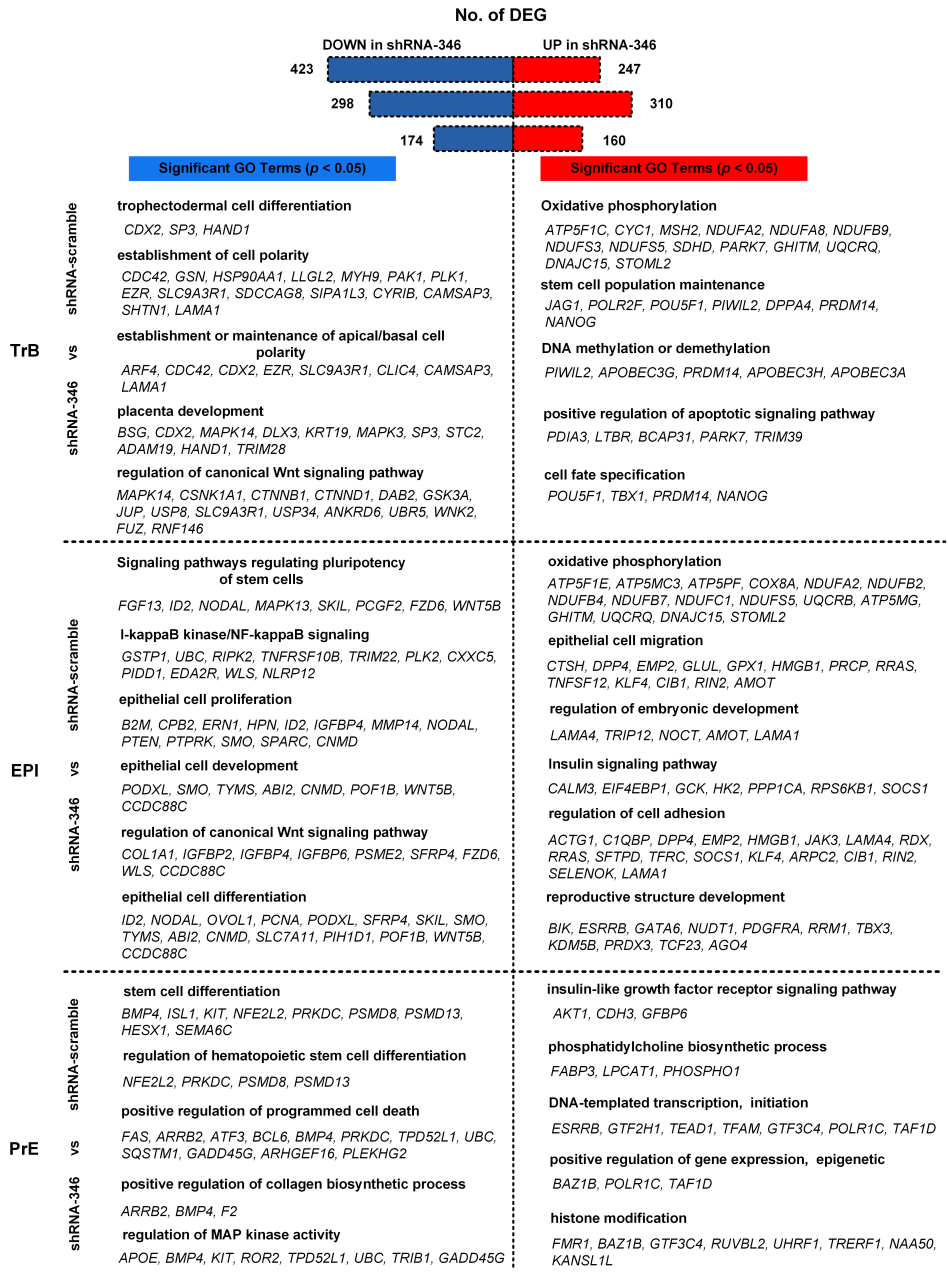
Differentially expressed genes (DEGs) and their corresponding gene ontology (GO) terms in TrB, EPI, and PrE from shRNA-346- or shRNA-scramble-treated embryos. DEGs were identified with function of FindMarkers in Seurat, test.use with DEseq2. *p*-value < 0.05, min.pct = 0.1. Significant upregulated and downregulated genes were used to identify enriched GO terms with the Metascape website (https://metascape.org/, accessed on 28 September 2021).

Next, we evaluated the DEGs (different expression genes) between shRNA-346 and shRNA-scramble embryonic cells. We noted that 247 and 423 genes were significantly upregulated and downregulated in shRNA-346-treated TrB cells when compared to shRNA-scrambled control (Figure 5). The upregulated genes were mainly associated with stem cell population maintenance, oxidative phosphorylation, DNA methylation or demethylation, positive regulation of apoptotic signaling pathway, and cell fate specification. The downregulated genes were mainly related to trophectodermal cell differentiation, the establishment of cell polarity, establishment or maintenance of apical/basal cell polarity, placenta development, and regulation of canonical Wnt signaling pathway (Table S3). These data suggest that MacERV6-LTR1a potentially regulates the development of TrB cells.

We also compared EPI cells treated with shRNA-346 and shRNA-scramble. We observed that 310 and 298 genes were significantly upregulated and downregulated in shRNA-346-treated EPI cells, respectively (Figure 5). Upregulated genes were mainly associated with oxidative phosphorylation, epithelial cell migration, regulation of embryonic development, insulin signaling pathway, regulation of cell adhesion, and reproductive structure development. Whereas downregulated genes were related to signaling pathways regulating pluripotency of stem cells, I-kappaB kinase/NF-kappaB signaling, epithelial cell proliferation, epithelial cell development, regulation of canonical Wnt signaling pathway, and epithelial cell differentiation (Table S3), these results suggest that MacERV6-LTR1a is required for regulation of the pluripotency of EPI cells.

We also compared PrE cells treated with shRNA-346 and shRNA-scramble. We noted that 160 and 174 genes were significantly upregulated and downregulated in shRNA-346-treated PrE cells, respectively (Figure 5). The upregulated genes were mainly enriched for insulin-like growth factor receptor signaling pathway, phosphatidylcholine biosynthetic process, DNA-templated transcription, initiation, positive regulation of gene expression, epigenetic, histone modification. Whereas downregulated genes were mainly associated with stem cell differentiation, regulation of hematopoietic stem cell differentiation, positive regulation of programmed cell death, positive regulation of collagen biosynthetic process, regulation of MAP kinase activity (Table S3). These data suggest that MacERV6-LTR1a mainly participates in the transcriptional activity and stem cell differentiation of early embryo development.

### 3.4. The Interaction between MacERV6-LTR1a and Pluripotency Factors *In Vitro*

Several LTR elements have been reported as tissue-specific enhancers or promoters in a variety of mammalian cell types, including early mouse embryos, placenta, pluripotent stem cells [29,35,62], and the initiation of transcriptional activity in specific cell lineages [37]. So, we hypothesized that MacERV6-LTR1a may act as a promoter or enhancer to recruit ICM, pre-EPI-specific TFs.

**Figure 6:**
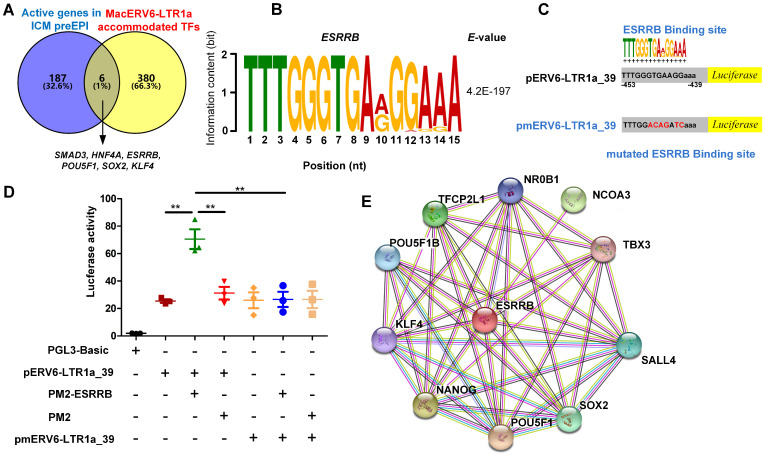
In vitro MacERV6-LTR1a interactions with pluripotency factors. (**A**) A Venn diagram showing overlapping transcription factors (TFs) specifically activated in ICM and pre-EPI cells and potentially recruited by MacERV6-LTR1a. Overall, 193 active genes in ICM and preEPI cells were reported in [52]. Additionally, 386 MacERV6-LTR1a accommodated TFs were predicted with MEME [60] and Tomtom [61]. (**B**) The motif of ESRRB in MacERV6-LTR1a and its E-value were predicted and calculated by MEME, respectively. The height of the nucleotide (Y-axis) is information content (in bit) and the X-axis is the position in the motif (nt). (**C**) ESRRB binding sites mutation in MacERV6-LTR1a.The MacERV6-LTR1a_39 element sequence that contained multiple pluripotency TF-binding sites have cloned into PGL3-basic vector and driven firefly luciferase activity (named pERV6-LTR1a_39). The nucleotide sequences of MacERV6-LTR1a_39, which the ESRRB binding site had mutated and cloned into the PGL3-basic vector (named pmERV6-LTR1a_39). (**D**) ESRRB interacting with MacERV6-LTR1a by luciferase assay in 293T cells. PM2-ESRRB, the PM2 vector containing coding sequence of ESRRB. pmERV6-LTR1a_39, pERV6-LTR1a_39, the plasmid contains luciferase reporter driven by ESRRB binding sites mutated MacERV6-LTR1a_39 and MacERV6-LTR1a_39, respectively. ** *p*-value < 0.01. Student’s *t*-test. (**E**) The protein–protein interaction network of ESRRB protein and others identified with the STRING web tool (https://string-db.org/, accessed on 28 September 2021).

To address this hypothesis, we identified motifs and predicted candidate TFs recruited by MacERV6-LTR1a. By integrating these candidate TFs with TFs specific for ICM and Pre-EPI cells, we identified *ESRRB*, *KLF4*, *POU5F1*, *SOX2*, *SMAD3*, and *HNF4A*, which were highly expressed in ICM and PreEPI cells, and may be recruited by MacERV6-LTR1a (Figure 6A,B and Figure S1A).

To further investigate the interaction between MacERV6-LTR1a and TFs, we performed luciferase assays. After transfecting the MacERV6-LTR1a-driven luciferase plasmid (pERV6-LTR1a_39) into 293T cells, we detected strong luciferase signals suggesting MacERV6-LTR1a had a strong promoter activity. Next, we co-transfected TF expressing vectors and pERV6-LTR1a_39 plasmid into 293T cells and observed that the *KLF4*, *POU5F1*, *SOX2*, *SMAD3*, and *HNF4A* co-transfections did not induce a significant increase in luciferase activity (Figure S1B–E). In contrast, the luciferase activity has significantly increased when added ESRRB, suggesting MacERV6-LTR1a recruited ESRRB. Since ESRRB induced the highest luciferase activity, we then mutated putative ESRRB binding sites in MacERV6-LTR1a to block ESRRB binding (pmERV6-LTR1a_39). We observed pmERV6-LTR1a_39 had lost their ability to enhance luciferase activity even in the presence of ESRRB (Figure 6C,D), suggesting ESRRB was recruited by MacERV6-LTR1a through the identified motif. Finally, we predicted functional associations between ESRRB and other proteins with STRING [63], and found that it is associated with self-renewal and pluripotency regulating genes, including KLF4, SOX2, NANOG, TFCP2L1, and POU5F1 (Figure 6E). These results may imply MacERV6-LTR1a recruited ESRRB to regulate gene expression, transcription activity, maintenance of self-renewal, and pluripotency of embryonic cells in an indirect way.

## 4. Discussion

The precise temporal development of embryonic cells is key to normal embryo development. At the beginning of the pre-implantation period, the embryo undergoes major transcriptional changes that establish a base for subsequent developmental phases. For example, FGF-4 ligands and FGF/ERK signaling are considered as the key cell-fate determination factors during this process in mice, but not in humans [64,65]. As mouse and primate embryogenesis are significantly diverse during successive stages, it is vital to investigate temporal development directly in monkey embryos.

In this study, we identified a large number of LTR elements, including HERVH, LTR7, LTR7B, LTR7-Rhe, LTR5 and LTR5-Hs, which are highly expressed in monkeys pre-implantation embryos but rapidly shutdown in post-implantation which was similar to mouse and human embryos [30,34]. A close relationship between LTR elements and embryonic development has been reported in human and mouse embryos [30,66,67]. However, it is unknown whether monkey-specific LTR elements are involved in the precise development of earlier pre-implantation embryos.

In humans, the embryo forms a blastocyst consisting of the ICM (inner cell mass), trophectoderm, and blastocoelic cavity at embryonic day 5-6 [68]. Similarly, the blastocysts of cynomolgus monkey can be observed at embryonic day 5-6 [52]. After blastocyst maturation in humans, the process of adhesion and implantation is occurring between embryonic days 7 and 8 [69], and cynomolgus monkey occurred in 7–9 days [45,52]. Lineage segregation occurred, but incompletely in human day 6, and cell fates of embryos became more fixed on day 7–9 [70]. While the cynomolgus monkeys began on day 7 [52], the lineage separation was not completed until day 10 [45]. Generally, the pregnancy period of cynomolgus monkeys is 24–30 weeks, while that of humans is 38–40 weeks. These results suggest that the early development of monkey embryos is different from that of human embryos.

In our research, we observed some monkey-specific LTRs (MacERV6-LTR, MacERVK1-LTR, MacERVK2-LTR, MacERV3-LTR, MacERV2-LTR, and MacERV1-LTR) were specifically expressed in blastocyst of pre-implantation stages and rapidly diminished in post-implantation stages. Even though blastocysts appeared morphologically identical between shRNA-scrambled and shRNA-346-treated embryos at day 7, changes in the differentiation of embryonic cells were noticed at the transcriptomic level. Importantly, we observed that knockdown of MacERV6-LTR1a can postpone the differentiation of TrB, EPI, and PrE cells.

MacERV6-LTR1a can recruit ESRRB, which plays an important role in the self-renewal, pluripotency of embryonic and trophoblast stem cells through different signaling pathways including FGF signaling pathway and Wnt signaling pathways [46,47,48,49,50,51]. In addition, ESRRB interacts with other important stem-cell TF, such as OCT4, NANOG, SOX2, KLF4, and TFCP2L1 (Figure 6E). We observed that *NANOG*, *POU5F1*, and *KLF4* were significantly highly expressed in TrB cells of shRNA-346 treated (Figure S2A–C, respectively), which may prevent TrB differentiation during embryonic development [71,72]. *TBX3* and *KLF4* are usually act as a transcriptional repressor in embryonic developmental processes, which highly expressed in EPI of shRNA-346 treated (Figure S2C,F, respectively), that means the gene relate differentiation may be inhibited in EPI cells of shRNA-346 treated embryos [73,74,75]. *TFCP2L1*, *SALL4*, and *NCOA3* play a key role in facilitates establishment, self-renewal, and transcriptional activities of embryonic stem cells, which have the tendency highly expressed in PrE of shRNA-346 treated (Figure S2D,E,G, respectively), suggesting that the changes of pluripotency in PrE may relate to MacERV6-LTR1a [75]. Thus, these might give us a new mechanism controlling early development in monkey embryos.

In this study, we observed that many LTRs of ERVs, including MacERV6-LTR1a, are activated in ICM and preEPI cells of monkey embryos, suggesting that other LTRs may involve in embryonic development via compensatory mechanisms. Therefore, it will be interesting to identify the function of other LTR elements during monkey embryo development in the future.

In summary, our results suggest that MacERV6-LTR1a is involved in precise gene regulation in the early developmental stages of monkey embryos. Because ERV6 is monkey specific, the activation of MacERV6-LTR in pre-implantation stages may contribute to different developmental patterns of monkey embryos.

## Data Availability

The scRNA-seq data generated in this study have been deposited at NCBI GEO database under accession number GSE182061.

## Supplementary Materials

The following are available online at https://www.mdpi.com/article/10.3390/cells10102710/s1 Data. Figure S1: MacERV6-LTR1a interactions with pluripotency factors. The specific TFs, such as *SMAD3*, *SOX2*, *KLF4*, *POU5F1* or *HNF4A*, interact with pERV6-LTR1a_39 elements in 293T cells by luciferase assay. Figure S2: Vinplots and comparisons of *NANOG*, *POU5F1*, *KLF4*, *TFCP2L1*, *SALL4*, *TBX3*, and *NCOA3* expression in TrB, EPI, and PrE cells which treated with shRNA-346 and shRNA-scramble lentivirus. Table S1: Short hairpin RNA (shRNA) primers targeting MacERV6-LTR1a and shRNA scrambled control primers. Table S2: Primers used to clone coding sequences of pluripotent genes and primers for qRT-PCR. Table S3: Differentially expressed genes (DEGs) and their corresponding gene ontology (GO) terms and Kyoto Encyclopedia of Genes and Genomes (KEGG) pathways in TrB, EPI, and PrE from shRNA-346- or shRNA-scramble-treated embryos.

## Abbreviations

The following abbreviations are used in this manuscript:

TF	Transcription factor
TrB	Trophectoderm
TE	Transposable Element
ICM	Inner cell mass
EPI	Epiblast
preEEPI	Pre-implantation early epiblast
preETE	Pre-implantation early trophectoderm
Pre-EPI	Pre-implantation epiblast
postE-EPI	Post-implantation early epiblast
postL-EPI	Post-implantation late epiblast
PrE	Primitive endoderm
ERV	Endogenous retroviruse
LTR	Long terminal repeat
hESC	Human embryonic stem cell
KRAB-ZFP	KRAB-containing zinc finger protein
rhFSH	Recombinant human follitropin alpha
rhCG	Recombinant human chorionic gonadotropin alpha
TALP	Tyrode’s albumin lactate pyruvate
BSA	Bovine serum albumin
MOR	Morula
DEGs	Differentially expressed genes
Gast1	Gastrulating cells 1
Gast2a	Gastrulating cells 2a
Gast2b	Gastrulating cells 2b
