# Supplementary material for: The Long Terminal Repeats of ERV6 Are Activated in Pre-Implantation Embryos of Cynomolgus Monkey
Source: Cells. 2021 Oct 9;10(10):2710. doi: 10.3390/cells10102710 (PMC8534818; doi:10.3390/cells10102710)
Supplement: Supplementary file 1 [file cells-10-02710-s001.zip › cells-1365745-SI/Supplementary Files/Figure S1.pdf]

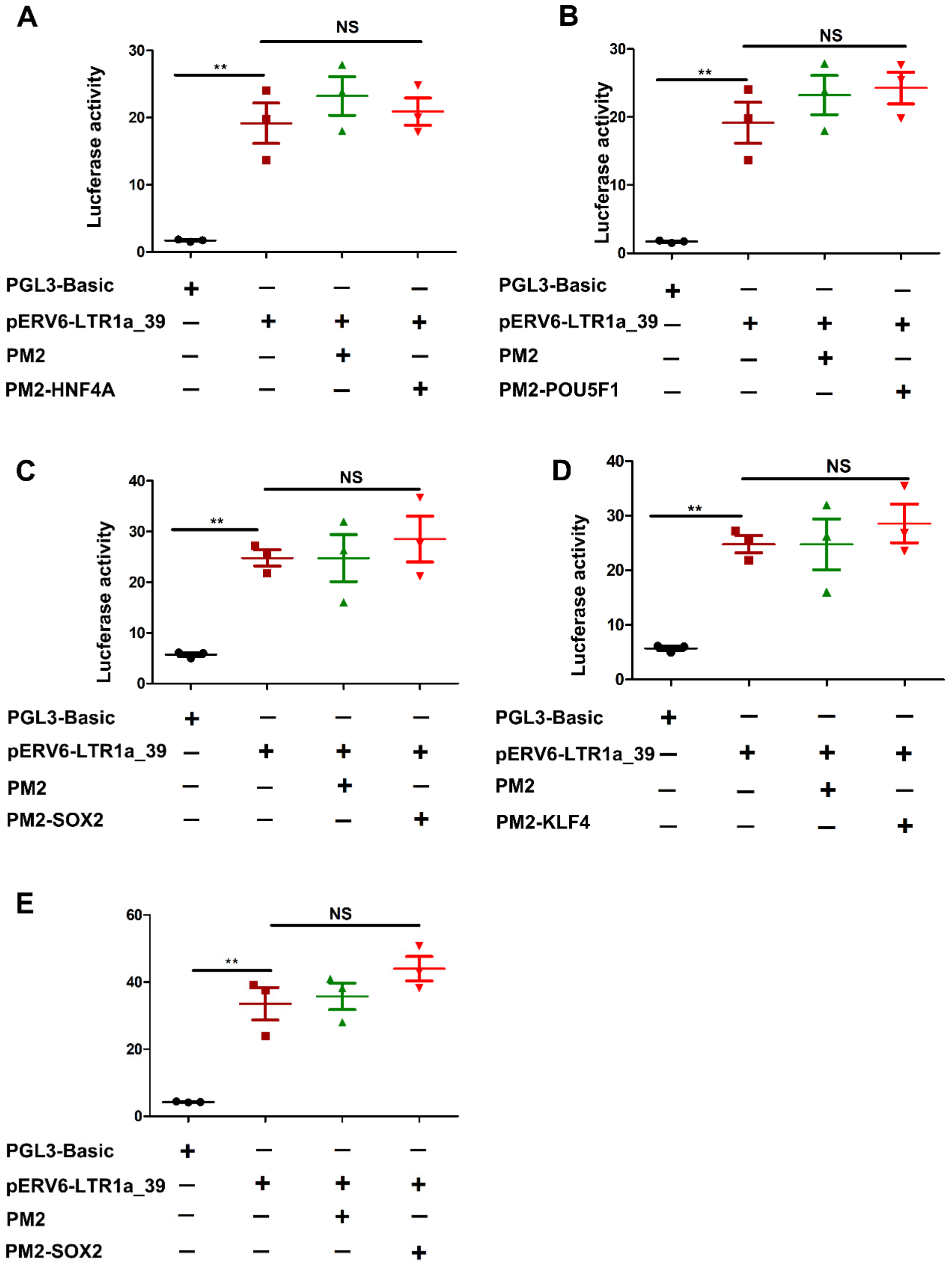

Figure S1 *In vitro* MacERV6-LTR1a interactions with pluripotency factors. (A-E) The efficiency of specific TFs, such as SMAD3, SOX2, KLF4, POU5F1 or HNF4A, interact with pERV6-LTR1a_39 elements in 293T cells by luciferase assay. The coding sequences of these genes were cloned into the PM2 expression vector. PGL3-basic: control plasmid without promoter, pERV6-LTR1a_39 plasmid containing the luciferase reporter driven by MacERV6-LTR1a_39, PM2: control plasmid without coding sequence of TFs. ***p-value* < 0.01. NS, not significant. Student's *t-test*.
