# Supplementary material for: The Long Terminal Repeats of ERV6 Are Activated in Pre-Implantation Embryos of Cynomolgus Monkey
Source: Cells. 2021 Oct 9;10(10):2710. doi: 10.3390/cells10102710 (PMC8534818; doi:10.3390/cells10102710)
Supplement: Supplementary file 1 [file cells-10-02710-s001.zip › cells-1365745-SI/Supplementary Files/Figure S2.pdf]

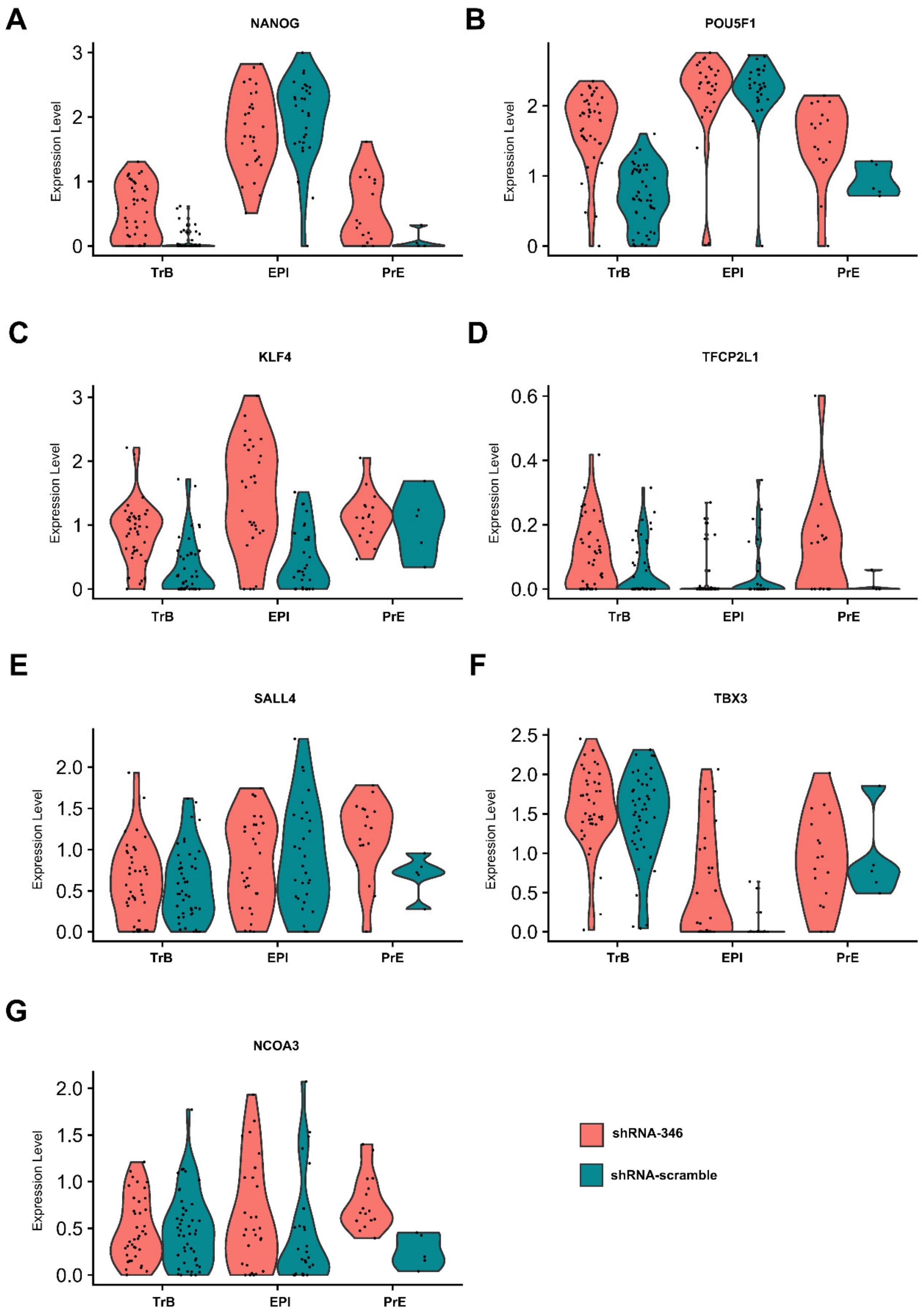


Figure S2 (A-G) Vinplots of *NANOG*, *POU5F1*, *KLF4*, *TFCP2L1*, *SALL4*, *TBX3*, and *NCOA3* expression in TrB, EPI, and PrE cells which treated with shRNA-346 and shRNA-scramble lentivirus.
